# Tegafur–Uracil Maintenance Therapy in Non-Metastatic Head and Neck Cancer: An Exploratory Systematic Review

**DOI:** 10.3390/curroncol32050286

**Published:** 2025-05-20

**Authors:** Hsu-Lin Lee, Po-Huang Chen, Tzu-Chuan Huang, Ren-Hua Ye, Yueng-Hsiang Chu, Jih-Chin Lee, Hong-Jie Jhou, Jia-Hong Chen

**Affiliations:** 1 Division of Hematology and Oncology, Department of Internal Medicine, Taichung Armed Forces General Hospital, Taichung 411228, Taiwan; 2 Division of Hematology and Oncology, Department of Internal Medicine, Tri-Service General Hospital, National Defense Medical Center, Taipei 114202, Taiwan; 3 Department of Otolaryngology–Head and Neck Surgery, Tri-Service General Hospital, National Defense Medical Center, Taipei 114202, Taiwan; 4 Department of Neurology, Changhua Christian Hospital, Changhua 500011, Taiwan; 5 Graduate Institute of Clinical Medicine, College of Medicine, Taipei Medical University; Taipei 11031, Taiwan

**Keywords:** tegafur–uracil, maintenance therapy, head and neck squamous cell carcinoma, nasopharyngeal carcinoma

## Abstract

Background: Tegafur–uracil (UFT), an oral fluoropyrimidine developed in Asia, has been investigated as a maintenance or adjuvant therapy in various malignancies. Its use in head and neck cancers, however, remains limited to small retrospective studies, primarily from East Asia. Given the need for cost-effective maintenance strategies in resource-limited settings, we conducted an exploratory systematic review to evaluate the clinical utility of UFT in non-metastatic head and neck squamous cell carcinoma (HNSCC) and nasopharyngeal carcinoma (NPC). Methods: We systematically searched PubMed, EMBASE, and Cochrane Library from inception through 1 May 2025 for retrospective cohort studies evaluating UFT after definitive therapy in non-metastatic HNSCC or NPC. Study selection followed PRISMA guidelines. Given the heterogeneity of included studies, we performed a structured narrative synthesis using the SWiM (Synthesis Without Meta-analysis) framework to summarize survival outcomes, treatment settings, and clinical contexts. Results: Seven retrospective studies (four HNSCC, three NPC) involving 508 patients were included. UFT was generally administered at 300–400 mg/day for 6–12 months. Across studies, UFT use was associated with favorable disease-free and overall survival trends in high-risk subgroups, including patients with extranodal extension and persistent EBV DNA. Treatment adherence and toxicity profiles were acceptable. Conclusions: While the evidence remains limited and heterogeneous, this review highlights recurring signals of benefit associated with UFT maintenance therapy in selected high-risk patients. Prospective trials are warranted to confirm these findings and better define a possible role of UFT in maintenance therapy in some advanced non-metastatic HNSCC and NPC.

## 1. Introduction

Recurrence in non-metastatic head and neck squamous cell carcinoma (HNSCC) and nasopharyngeal carcinoma (NPC) remains a major concern, particularly within the first two years after treatment completion [1,2]. Most patients enter a surveillance phase without active treatment [3]. In some other malignancies, such as colorectal and breast cancer, well-tolerated maintenance therapy—defined as the administration of lower-intensity treatment after completion of curative therapy—has demonstrated benefits in prolonging disease control while maintaining tolerability [4,5].

Tegafur–uracil (UFT), an oral combination of a 5-fluorouracil (5-FU) prodrug (tegafur) and a dihydropyrimidine dehydrogenase inhibitor (uracil), provides sustained tumor exposure to 5-FU with relatively low systemic toxicity [6,7]. In addition to its cytotoxic effects via thymidylate synthase inhibition and RNA disruption [8,9], preclinical data suggest that UFT may exert anti-angiogenic activity and modulate the tumor immune microenvironment by reducing myeloid-derived suppressor cells [10]. These mechanisms support its potential role in controlling minimal residual disease following curative treatment.

Seven small, non-randomized retrospective cohort studies have explored UFT as maintenance therapy in head and neck cancers. There are three reports in NPC (n = 558) in patients with detectable post-treatment plasma Epstein–Barr virus (EBV) DNA, a known predictor of recurrence [11,12]. There are four reports in high-risk HNSCC, such as those with extranodal extension or advanced stage [13,14]. The magnitude of benefit, ideal treatment duration, and target populations remain undefined. We undertook a systematic review of available studies evaluating UFT maintenance therapy in non-metastatic NPC and HNSCC. Our aim was to introduce and synthesize existing evidence on this underutilized, low-cost agent and to support consideration for future prospective validation.

## 2. Materials and Methods

This systematic review incorporated structured qualitative analysis following the Synthesis Without Meta-analysis (SWiM) framework to accommodate heterogeneity in study designs and cancer subtypes.

### 2.1. Search Strategy and Study Selection

A systematic literature search was conducted in PubMed, EMBASE, and Cochrane Library databases from inception until 1 May 2025. The search strategy combined terms related to head and neck cancer, NPC, UFT, and maintenance therapy. Information was collected regarding study design, sample size, recruitment period, follow-up duration, patient demographics, disease characteristics, treatment details, and survival outcomes. The quality of included studies was assessed using the Risk of Bias in Non-Randomized Studies of Interventions (ROBINS-I, version 1) tool. The study search strategy and literature identification methods are illustrated in Supplement S1.

### 2.2. Eligibility Criteria

Studies were eligible for inclusion if they met the following criteria:

1. Study design: randomized controlled trials, prospective cohort studies, or retrospective cohort studies with a comparator group

2. Population: adult patients with non-metastatic head and neck cancers, including NPC and head and HNSCC.

3. Intervention: oral UFT as maintenance therapy after definitive treatment (surgery, radiotherapy, concurrent chemoradiotherapy).

4. Comparison: no maintenance therapy or standard follow-up after definitive treatment

5. Outcomes: reported data on overall survival (OS), progression-free survival (PFS), disease-free survival (DFS), or equivalent endpoints with hazard ratios (HRs) and 95% confidence intervals (CIs) or sufficient information to estimate these parameters.

Studies were excluded if they (1) included patients with metastatic disease, (2) lacked a comparison group, (3) provided insufficient outcome data, (4) were single-arm studies, or (5) focused solely on quality of life or toxicity outcomes without survival data.

### 2.3. Data Extraction and Quality Assessment

Two investigators independently extracted data using standardized forms. The following information was collected: first author, publication year, country, study design, sample size, recruitment period, follow-up duration, patient demographics, disease characteristics, treatment details, and survival outcomes. The quality of included studies was assessed using the ROBINS-I tool, as all included studies were cohort studies.

### 2.4. Critical Appraisal Using SWiM Guidelines

A SWiM approach is a transparent, reproducible method for summarizing evidence when meta-analysis is inappropriate due to factors such as small numbers or variability in populations, interventions, or outcome measures. We used a SWiM framework to conduct a structured qualitative appraisal of the included studies, systematically assessing how (1) studies were grouped for synthesis (by tumor type and risk profile), (2) intervention effects were standardized for interpretability, and (3) variations in study design and outcome reporting were handled.

Specifically, we reviewed each study’s clinical rationale, implementation of UFT maintenance therapy (e.g., timing, duration, and dosing), outcome definitions, and key findings. These components were then, as recommended by SWiM guidance, summarized across four dimensions: (1) study rationale, (2) what was done, (3) interpretation, and (4) clinical meaning.

## 3. Results

### 3.1. Search Results and Study Selection

From PubMed, Embase, and the Cochrane Library (search date: 1 May 2025): after removing 92 duplicates, 302 titles and abstracts were screened, with 276 excluded due to irrelevance or ineligible study design. Of the 26 full-text articles reviewed, 19 were excluded for reasons including metastatic populations, insufficient survival data, or lack of a comparison group. The remaining seven retrospective cohort studies met the inclusion criteria and were included in both the qualitative and quantitative syntheses. The study selection process is illustrated in Supplement S2. All seven were rated as having “serious” risk of bias by ROBINS-I, primarily due to confounding and selection bias. The risk of bias in outcome measurement and reporting was generally low. Detailed assessments are provided in Supplement S3.

This narrative synthesis highlights that while study designs were heterogeneous, the clinical rationale for UFT use was consistent, targeting high-risk, non-metastatic patients who remain vulnerable to recurrence despite completion of definitive therapy.

### 3.2. Head and Neck Squamous Cell Carcinoma: A Summary of Four Studies

Table 1 presents a synthesis of four included studies on HNSCC, using the SWiM framework across four analytical dimensions: (1) study rationale, (2) what was done, (3) interpretation of findings, and (4) clinical meaning. Key clinical characteristics and outcomes are also summarized. A detailed SWiM-based critical appraisal is available in Supplement S4.

The primary rationale across four studies centered on the hypothesis that metronomic oral UFT could reduce recurrence risk following definitive therapy in high-risk patients. For instance, Huang et al. [13] and Huang et al. [14] investigated postoperative UFT in oral cavity squamous cell carcinoma, particularly in patients with extranodal extension (pENE+), a well-recognized risk factor for distant failure. Yeh et al. [16] evaluated UFT maintenance following CCRT in locally advanced HNSCC and reported improvements in OS, DFS, and distant metastasis-free survival (DMFS), while Lien [15] explored biomarker-guided selection using cortactin expression in hypopharyngeal cancer, suggesting a potential role for UFT in molecularly defined subgroups. These studies consistently reported favorable survival trends and acceptable toxicity profiles, although all were limited by retrospective design and lack of prospective biomarker validation.

The mean or median patient age ranged from 50 to 56 years, and they were mainly male patients (90% to 98%). The proportion of patients with good performance status (ECOG 0–1) ranged from 67% to 100%. Most patients had advanced T-stage (T3–T4: 47% to 74%), and many had advanced nodal disease, with N3 involvement reported in up to 92% in selected subgroups.

The HNSCC studies reported favorable outcomes associated with UFT use. Huang et al. [13] demonstrated improved 3-year OS and DFS with UFT. Huang et al. [14] showed reductions in distant metastasis and improved OS (HR = 0.31) and EFS (HR = 0.45) among pENE+ patients. Lien et al. [15] found that UFT significantly prolonged OS and RFS in cortactin-positive patients but not in cortactin-negative ones. Yeh et al. [16] observed improved DFS (HR = 0.51) and DMFS (HR = 0.57), with longer UFT use associated with better outcomes.

### 3.3. Nasopharyngeal Carcinoma: A Summary of Three Studies

Table 2 summarizes the characteristics of the three included studies on NPC, structured using the SWiM framework. Key clinical characteristics and outcomes are also summarized. A detailed SWiM-based critical appraisal is available in Supplement S4.

The rationale for UFT therapy was even more focused, targeting patients at high risk of relapse based on either clinical staging (e.g., stage IV non-metastatic disease) or persistently detectable post-treatment EBV DNA, a molecular marker strongly associated with recurrence and metastasis. Chen et al. [11], Liu et al. [17], and Twu et al. [12] each employed UFT as maintenance or adjuvant therapy in this setting, reporting substantial improvements in OS and PFS, particularly among biomarker-positive patients. The use of UFT in these studies was framed as a feasible, pragmatic alternative to more resource-intensive therapies, particularly in regions where EBV DNA monitoring or immunotherapy may not be readily available.

The mean age ranged from 44 to 49 years, and 67% to 83% were male, with a Karnofsky performance status ≥ 80% in 84% to 90% of patients. T3–T4 disease accounted for 71% to 87%, while N3 disease ranged from 27% to 39%. Chen et al. [11] reported higher 5-year OS (92% vs. 58%) and DFS (73% vs. 36%) in the UFT group. Liu et al. [17] found improved OS (81% vs. 67%) and DMFFS, particularly in patients with undetectable post-RT EBV DNA. Twu et al. [12] demonstrated better OS and MFS in patients with elevated post-RT EBV DNA who received UFT.

## 4. Discussion

This exploratory review summarizes the available retrospective data on UFT as maintenance therapy in non-metastatic head and neck cancers. All studies varied in patient selection, dosing strategies, and clinical settings. Several reported UFT use improved disease-free and overall survival rates. These signals were most evident in high-risk subgroups, such as patients with extranodal extension or persistent post-treatment EBV DNA. While not definitive, the consistency of findings across heterogeneous cohorts suggests UFT may be a candidate for further prospective evaluation, particularly in resource-limited settings where access to newer (and more expensive) agents remains a challenge.

### 4.1. Comparison with Other Maintenance Therapies: Oral Fluoropyrimidines and Targeted Approaches

Compared to other oral fluoropyrimidines such as capecitabine and S-1, UFT may offer a comparable maintenance strategy for non-metastatic head and neck cancers, with distinct advantages in tolerability, as UFT has a lower incidence of hand–foot syndrome and hematologic toxicity. In a Chinese phase III trial, one year of capecitabine maintenance following CCRT in locoregionally advanced NPC improved three-year failure-free survival [18]. Similarly, the ACTS-HNC trial showed that one year of S-1 maintenance yielded three-year DFS and OS rates comparable to historical UFT data in HNSCC [19]. Our findings align with these results, suggesting that UFT may be a viable alternative in clinical scenarios where toxicity and cost are key concerns.

Other maintenance strategies, such as EGFR-targeted therapy and immune checkpoint inhibitors, have been explored. Cetuximab has shown benefit in small trials but is limited by toxicity and lack of phase III confirmation [20]. PD-1 inhibitors have demonstrated encouraging results in post-CCRT NPC, especially in patients with elevated EBV DNA [21]; however, their role in HNSCC remains uncertain based on negative or inconclusive trial outcomes [22,23].

### 4.2. Implementation Challenges in Clinical Practice

Implementing UFT maintenance therapy in clinical practice presents practical and logistical challenges. Patient selection remains a key consideration. In the reviewed studies, UFT was generally administered following definitive therapy in patients at elevated risk of recurrence, such as those with T3–T4 disease, extranodal extension, or elevated Epstein–Barr virus DNA in NPC [24]. However, no consensus criteria currently define which patients are most suitable for UFT maintenance, and prospective studies are needed to better guide risk-adapted application.

Toxicity monitoring is another factor. UFT is typically associated with mild adverse events, including low-grade gastrointestinal symptoms (e.g., nausea) and myelosuppression (e.g., anemia) [25]. Although serious toxicities were not reported in the included studies, cumulative low-grade events over extended periods may reduce adherence. The ACTS-HNC trial, for instance, reported that only 58.3% of patients completed the planned one-year course of UFT, underscoring the importance of patient education and supportive care to maintain compliance [19].

Moreover, despite its favorable toxicity profile, oral administration, and cost-effectiveness, UFT’s availability remains largely confined to East Asia. Broader access may depend on local regulatory approvals and inclusion in national formularies. At present, UFT is not incorporated into major international clinical guidelines and should be regarded as investigational.

### 4.3. Limitations

This review has several limitations. First, all included studies were retrospective cohorts, and most were assessed as having a serious risk of bias using the ROBINS-I tool, particularly due to confounding and selection bias. The absence of randomized controlled trials limits the ability to establish causal inference. Second, the small number of studies and their methodological heterogeneity precluded formal assessments of publication bias and limited the feasibility of subgroup or sensitivity analyses. Therefore, instead of attempting pooled statistical estimates, we adopted a structured narrative synthesis based on the SWiM framework to better accommodate the clinical and design variability across studies. Third, differences in UFT dose (300–400 mg/day), treatment duration (6–12 months), and timing of administration (postoperative, post-radiotherapy, or post-CCRT) limit comparability and generalizability. Lastly, the follow-up durations, while adequate for mid-term outcome assessment, may not fully capture late recurrence or toxicity. Most studies were conducted in East Asia, which may also limit geographic generalizability to other populations and practice settings.

In addition to the geographic limitations discussed next, variability in UFT administration further complicates efforts to standardize protocols for randomized trials. UFT was used either as a daily dose of 300 or 400 mg. The intended duration of maintenance therapy also varied, ranging from 6 to 12 months, though most studies aimed for a year. In addition, UFT was applied in diverse settings, including post-radiotherapy in NPC and postoperative adjuvant maintenance following CCRT in HNSCC. These differences may influence adherence and efficacy and highlight the need for protocol-standardized trials.

### 4.4. Geographic Limitations, Global Relevance, and Barriers to Randomized Trials

All included studies were conducted in East Asia, primarily in Taiwan and Japan, reflecting regional treatment practices and drug accessibility. UFT, developed by Japanese pharmaceutical companies, has long been used in Asia but remains unavailable or unapproved in many Western countries. Consequently, oral fluoropyrimidines such as capecitabine and S-1 are more commonly studied and prescribed in Western practice. This geographic and regulatory limitation partly explains the lack of UFT-based trials in Europe or North America. However, this underscores rather than diminishes the need for rigorous synthesis of the available evidence. By consolidating real-world data from Asian populations, we aim to improve global awareness of UFT as a low-cost, orally administered agent with potential applicability beyond its current regional confines. Such evidence may help guide future clinical trials, policy considerations, and access in underserved populations where newer agents may not be readily available.

Despite the encouraging results seen in retrospective studies, conducting randomized control trials (RCTs) for UFT maintenance remains challenging. These challenges include limited commercial incentives to study an older, generic agent; logistical complexities in long-term follow-up; and low prioritization of maintenance strategies in funding agendas. Nevertheless, the reproducible signals of benefit observed in both HNSCC and NPC support the need for pragmatic RCTs in regions where UFT is accessible and affordable.

## 5. Conclusions

This exploratory systematic review provides a structured synthesis of available evidence regarding the use of UFT as maintenance or adjuvant therapy in non-metastatic HNSCC and NPC. In four and three retrospective cohort studies, UFT was associated with improved survival outcomes in selected high-risk populations. However, the retrospective nature of all studies, regional concentration of data, and heterogeneity in treatment protocols limit the strength of inference.

By applying the SWiM framework, this review offers a cautious interpretation of clinical signals. Future prospective, randomized, multicenter trials are warranted to clarify the role of UFT, standardize treatment protocols, and determine its potential integration into global clinical practice. In the absence of such trials, the available evidence may justify consideration of UFT in carefully selected, closely monitored high-risk patients, particularly in settings where alternative maintenance strategies are unavailable.

## Figures and Tables

**Table 1:** Head and neck squamous carcinoma: a summary based on the SWiM framework for narrative synthesis and characteristics.

The SWiM Framework for Narrative Synthesis							
Study	Study Rationale		What Was Done		Interpretation		Meaning
Huang (2021) [13] n = 93	Maintenance therapy may improve survival.		UFT 400 mg/d × 1 year vs. control post-CCRT; OS, DFS, DMFS; statistical: KM, log-rank, Cox.		3 years/5 years OS, DFS, DMFS independently predicted OS; tolerable AEs (anorexia).		UFT may reduce recurrence/metastasis; safe.
Huang (2022) [14] n = 103	Maintenance therapy may reduce metastasis and improve survival.		UFT 1 year vs. observation post-CCRT; OS, EFS, DMFS.		UFT reduced distant metastasis; better OS in relapsed UFT pts; mild toxicity.		Supports UFTm for high-risk pENE+ OCSCC; attractive due to oral route/toxicity.
Lien (2023) [15] n = 157	UFT may benefit in cortactin-positive patients.		UFT use by physician; RFS, OS; analysis by KM, Cox.		UFT improved OS/RFS only in cortactin+ pts.		UFT may benefit cortactin+ HPC; biomarker-stratified strategy proposed.
Yeh (2021) [16] n = 240	Metronomic UFT may improve OS, DFS, and DMFS.		UFT 100–400 mg/d for 3–12 months vs. no UFT post-CRT; OS, DFS, DMFS; multivariate/subgroup analyses.		UFT prolonged OS, DFS, DMFS; DFS; AEs (nausea, mucositis).		Safe and potentially effective for high-risk HNSCC post-CRT.
**Characteristics of Head and Neck Squamous Carcinoma**							
**Study**	**Cancer** **Type**	**Stage**	**Mean Age** **(Year** **s)**	**Gender** **(Male%)**	**ECOG PS** **(0–1)%**	**Primary Results** **UFT** **vs. Non-UFT**	**Subgroup Analyses** **UFT** **vs. Non-UFT**
Huang 2021 [13]	OC	IVa-IVb	UFT: 52 Non-UFT: 57	UFT: 90% Non-UFT: 88%	UFT: 92% Non-UFT: 67%	3-year OS: 75% vs. 48%, *p* = 0.001 5-year OS: 45% vs. 44%, *p* = 0.016 3-year DFS: 53% vs. 35%, *p* = 0.011 5-year DFS: 40% vs. 31%, *p* = 0.018	3-year DMFS: 64% vs. 43%, *p* = 0.004 5-year DMFS: 42% vs. 39%, *p* = 0.02
Huang 2022 [14]	OC	IVa-IVb	UFT: 50 Non-UFT: 50	UFT: 91% Non-UFT: 92%	100% (all ECOG PS 1)	2-year DMR: 26% vs. 44% OS: HR 0.31, *p* < 0.001 EFS: HR 0.45, *p* = 0.009 DMF: HR 0.47, *p* = 0.035	RR: 36% vs. 56%, *p* = 0.042 Median OS in relapsed patients: 21.0 vs. 11.0 months (*p* < 0.001)
Lien 2023 [15]	HPx	25% III, 75% IVa-IVb	Median: 54	98% (both groups)	Not reported	Cortactin expression: 1. Median RFS: 10.2 months (cortactin+) vs. 86.7 months (–), *p* < 0.001 2. Median OS: 16.9 months (cortactin+) vs. 93.4 months (–), *p* < 0.001	1. UFT effect in cortactin (+): (1) RFS: 13.6 vs. 7.0 months, *p* = 0.006 (2) OS: 24.0 vs. 10.0 months, *p* < 0.001 2. UFT effect in cortactin (–): No statistically significant difference in RFS or OS
Yeh 2021 [16]	OC, OPx, HPx	21% III, 79% IVa-IVb	UFT: 56 Non-UFT: 54	UFT: 98% Non-UFT: 97%	Not reported	1. OS: Not reached vs. 54.1 months, *p* = 0.008, HR = 0.57, *p* = 0.073 2. DFS: 54.5 vs. 34.4 months; HR = 0.51; *p* = 0.006) 3. DMFS: HR = 0.57, *p* = 0.019	Longer treatment duration with UFT (>6 months) may improve OS, DFS, and DMFS.

OCSCC: oral cavity squamous cell carcinoma; CCRT: concurrent chemoradiotherapy; UFT: tegafur–uracil; NPC: nasopharyngeal carcinoma; RCT: randomized control trial; PFS: progression-free survival; OS: overall survival; EFS: event-free survival; DFS: disease-free survival; DMFS: distant metastasis free survival; DMFFS: distant metastasis failure-free survival; KM: Kaplan–Meier, HR: hazard ratio; AEs: adverse events; pENE: pathologic extranodal extension; HPC: hypopharyngeal cancer; RFS: recurrence-free survival; QoL: quality of life; AdjCT: adjuvant chemotherapy; MFS: metastasis-free survival; RR: relapse rate; DMF: distal metastatic failure; OC: oral cavity; OPx: oropharynx; HPx: hypopharynx.

**Table 2:** Nasopharyngeal carcinoma: a summary based on the SWiM framework for narrative synthesis and characteristics.

The SWiM Framework for Narrative Synthesis							
Study	Study Rationale		What Was Done		Interpretation		Meaning
Chen (2019) [11] n = 70	Maintenance therapy may improve survival.		UFT 400 mg/d × 12 months vs. observation after CR post-CCRT; OS, DFS, AE.		UFT independently predicted improved OS/DFS; well-tolerated.		UFT maintenance improves OS and DFS; good tolerance; feasible for high-risk NPC.
Liu (2017) [17] n = 403	UFT could improve OS and PFS.		UFT (2 caps BID × 12 months) vs. no AdjCT; OS, PFS, DMFFS.		OS, PFS, DMFFS improved.		UFT prolongs OS and reduces metastasis.
Twu (2014) [12]	Biomarker-guided UFT AdjCT might improve outcomes.		UFT (2 caps BID × 12 months) vs. no treatment; OS, PFS, MFS.		OS, PFS, and MFS improved; mild toxicity.		UFT improves outcomes in EBV DNA-positive patients.
**Characteristics of** **Nasopharyngeal Carcinoma**							
**Study**	**Cancer** **Type**	**Stage**	**Mean Age** **(Years)**	**Gender** **(Male%)**	**ECOG PS** **(0–1)%**	**Primary Results** **UFT** **vs. Non-UFT**	**Subgroup Analyses** **UFT** **vs. Non-UFT**
Chen 2019 [11]	NPC	IVa-IVb	UFT: 49 Non-UFT: 44	83% (58/70)	UFT: 81% Non-UFT: 70%	5-year OS: 91.89% vs. 57.58%; *p* = 0.004 5-year DFS: 72.97% vs. 36.36%; *p* = 0.007 Multivariate analysis: OS (HR = 0.215; *p* = 0.03) DFS (HR = 0.366; *p* = 0.02)	-
Liu 2016 [17]	NPC	II-IV	UFT: 47 Non-UFT: 46	UFT: 75% Non-UFT: 74%	Karnofsky ≥ 80%: UFT 84% Non-UFT 90.0%	5-year OS: 80.5% vs. 66.7% 5-year PFS: 70.5% vs. 59.4% DMFFS: 82.1% vs. 68.5%	Patients with undetectable post-RT plasma EBV DNA (n = 327): OS (HR 0.59; *p* = 0.0071) and DMFFS (HR 0.61; *p* = 0.0481)
Twu 2014 [12]	NPC	IIB-IVB (AJCC 1997)	Both groups: 49	Adjuvant: 79% Control: 67%	Karnofsky ≥ 80%: 88%	5-year OS: 71.6% vs. 28.7% 5-year PFS: 62.9% vs. 28.7% 5-year MFS: 71.9% vs. 34.6%	UFT group had better OS (*p* = 0.0039) and MFS (*p* = 0.0232)

ECOG PS: Eastern Cooperative Oncology Group Performance Status; UFT: tegafur–uracil; NPC: nasopharyngeal carcinoma; OS: overall survival; PFS: progression-free survival; DFS: disease-free survival; DMFS: distant metastasis-free survival; DMFFS: distant metastasis failure-free survival; HR: hazard ratio; EFS: event-free survival; DMF: distant metastasis failure; RFS: recurrence-free survival; RT: radiation therapy; MFS: metastasis-free survival; AJCC: American Joint Committee on Cancer.

## Data Availability

The data presented in this study are available on request from the corresponding author.

## Supplementary Materials

The following supporting information can be downloaded at https://www.mdpi.com/article/10.3390/curroncol32050286/s1, Supplement S1: Search Strategy and Literature Identification Methods; Supplement S2: Flow Diagram of Literature Selection Process for Systematic Review; Supplement S3: Risk of Bias Assessment for Included Studies; Supplement S4: SWiM-Based Critical Appraisal.
