# Supplementary material for: Tegafur–Uracil Maintenance Therapy in Non-Metastatic Head and Neck Cancer: An Exploratory Systematic Review
Source: Curr Oncol. 2025 May 20;32(5):286. doi: 10.3390/curroncol32050286 (PMC12109691; doi:10.3390/curroncol32050286)
Supplement: Supplementary file 1 [file curroncol-32-00286-s001.zip › curroncol-3574237-supplementary.pdf]

Supplementary material

Tegafur-Uracil Maintenance Therapy in Non-Metastatic Head and Neck Cancer: An Exploratory Systematic Review

Contents

Supplement S1: Search Strategy and Literature Identification Methods

Supplement S2: Flow Diagram of Literature Selection Process for Systemic Review.

Supplement S3: Risk of Bias Assessment for Included Studies

Supplement S4: SWiM-Based Critical Appraisal

Supplement S1: Search Strategy and Literature Identification Methods
PubMed:
((("Head and Neck Neoplasms"[Mesh] OR "Nasopharyngeal Neoplasms"[Mesh] OR "head and neck"[Title/Abstract] OR "head neck"[Title/Abstract] OR "nasopharyn*"[Title/Abstract] OR "oral cancer*"[Title/Abstract] OR "oropharyn*"[Title/Abstract] OR "hypopharyn*"[Title/Abstract] OR "laryn*"[Title/Abstract] OR "NPC"[Title/Abstract] OR "HNSCC"[Title/Abstract] OR "SCCHN"[Title/Abstract])) AND ("Tegafur"[Mesh] OR "UFT"[Title/Abstract] OR "tegafur"[Title/Abstract] OR "5-fluoro-2'-deoxyuridine"[Title/Abstract] OR "Fluorodeoxyuridine"[Title/Abstract] OR "Floxuridine"[Title/Abstract] OR "Futraful"[Title/Abstract] OR "Tegafur-Uracil"[Title/Abstract] OR "uracil tegafur"[Title/Abstract] OR "Uftoral"[Title/Abstract])) AND ("Maintenance"[Title/Abstract] OR "maintainence"[Title/Abstract] OR "maintain*"[Title/Abstract] OR "continuation"[Title/Abstract] OR "consolidation"[Title/Abstract] OR "adjuvant"[Title/Abstract] OR "Drug Administration Schedule"[Mesh] OR "sequential"[Title/Abstract] OR "long term"[Title/Abstract] OR "follow up"[Title/Abstract])

EMBASE:
('head and neck tumor'/exp OR 'nasopharynx cancer'/exp OR 'head and neck':ti,ab OR 'head neck':ti,ab OR 'nasopharyn*':ti,ab OR 'oral cancer*':ti,ab OR 'oropharyn*':ti,ab OR 'hypopharyn*':ti,ab OR 'laryn*':ti,ab OR 'npc':ti,ab OR 'hnscc':ti,ab OR 'scchn':ti,ab) AND ('tegafur'/exp OR 'tegafur plus uracil'/exp OR 'fluoropyrimidine'/exp OR 'uft':ti,ab OR 'tegafur':ti,ab OR '5-fluoro-2-deoxyuridine':ti,ab OR 'fluorodeoxyuridine':ti,ab OR 'floxuridine':ti,ab OR 'futraful':ti,ab OR 'tegafur-uracil':ti,ab OR 'uracil tegafur':ti,ab OR 'uftoral':ti,ab) AND ('maintenance therapy'/exp OR 'maintenance':ti,ab OR 'maintainence':ti,ab OR 'maintain*':ti,ab OR 'continuation':ti,ab OR 'consolidation':ti,ab OR 'adjuvant':ti,ab OR 'drug administration'/exp OR 'sequential':ti,ab OR 'long term':ti,ab OR 'follow up':ti,ab)

Cochrane:
(MeSH descriptor: [Head and Neck Neoplasms] explode all trees OR MeSH descriptor: [Nasopharyngeal Neoplasms] explode all trees OR "head and neck":ti,ab,kw OR "head neck":ti,ab,kw OR "nasopharyn*":ti,ab,kw OR "oral cancer*":ti,ab,kw OR "oropharyn*":ti,ab,kw OR "hypopharyn*":ti,ab,kw OR "laryn*":ti,ab,kw OR "NPC":ti,ab,kw OR "HNSCC":ti,ab,kw OR "SCCHN":ti,ab,kw) AND (MeSH descriptor: [Tegafur] explode all trees OR "UFT":ti,ab,kw OR "tegafur":ti,ab,kw OR "FT":ti,ab,kw OR "5-fluoro-2'-deoxyuridine":ti,ab,kw OR "Fluorodeoxyuridine":ti,ab,kw OR "Floxuridine":ti,ab,kw OR "Futraful":ti,ab,kw OR "Tegafur-Uracil":ti,ab,kw OR "uracil tegafur":ti,ab,kw OR "Uftoral":ti,ab,kw) AND ("Maintenance":ti,ab,kw OR "maintainence":ti,ab,kw OR "maintain*":ti,ab,kw OR "continuation":ti,ab,kw OR "consolidation":ti,ab,kw OR "adjuvant":ti,ab,kw OR MeSH descriptor: [Drug Administration Schedule] explode all trees OR "sequential":ti,ab,kw OR "long term":ti,ab,kw OR "follow up":ti,ab,kw)

PubMed Search (2025/05/01): 229 results

Embase Search (2025/05/01): 124 results

Cochrane Library Search (2025/05/01): 44 results

Supplement S2: Flow Diagram of Literature Selection Process for Systemic Review.

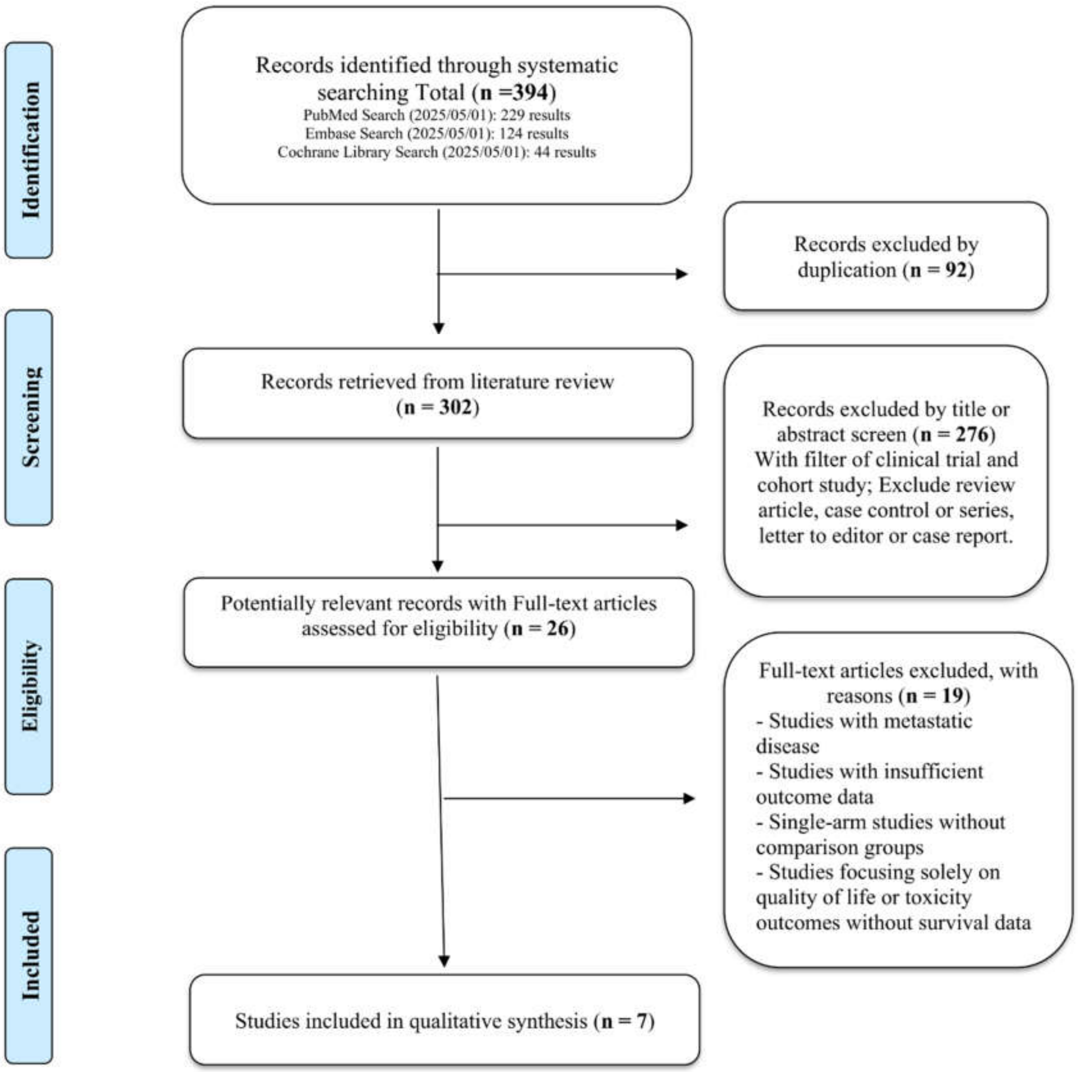

Flowchart showing the identification, screening, eligibility assessment, and final inclusion of studies according to PRISMA guidelines.

Supplement S3 Risk of Bias Assessment for Included Studies

ROBINS-I for non-randomized studies of interventions

Study	Confounding	Selection of participants	Classification of interventions	Deviations from intended interventions	Missing data	Measurement of outcomes	Selection of reported result	Overall bias
**Head and neck squamous cell carcinoma**								
Huang 2021	Serious	Moderate	Low	Moderate	Low	Low	Low	Serious
Huang 2022	Serious	Moderate	Low	Moderate	Low	Low	Low	Serious
Lien 2023	Serious	Moderate	Low	Low	Low	Low	Low	Serious
Yeh 2021	Serious	Serious	Moderate	Moderate	Low	Moderate	Low	Serious
**Nasopharyngeal carcinoma**								
Chen 2019	Serious	Serious	Moderate	Moderate	Low	Moderate	Low	Serious
Liu 2016	Serious	Serious	Moderate	Moderate	Low	Moderate	Low	Serious
Twu 2014	Serious	Moderate	Low	Low	Low	Moderate	Low	Serious

ROBINS-I Assessment for Huang et al. (2021)

1. Bias due to confounding

- **Rating: Serious risk**
- **Rationale**: This was a retrospective study where "physicians prescribed the drug based on their clinical judgment." Significant baseline differences exist, including age (p=0.025), tumor site distribution (p=0.0052), and ECOG performance status (p=0.0378). The UFT group had better performance status with 92% having ECOG 0-1 vs. 66.66% in the control group, which is an important prognostic factor that likely influenced outcomes independently of treatment.

2. Bias in selection of participants

- **Rating: Moderate risk**
- **Rationale**: Patients were selected from a database with clear inclusion criteria. However, selection for UFT treatment was based on physician discretion rather than randomization, potentially creating selection bias favoring healthier patients for UFT treatment.

3. Bias in classification of interventions

- **Rating: Low risk**
- **Rationale**: The intervention was clearly defined (UFT 400mg daily divided into two doses, 7 days a week for 1 year), and the comparison groups were clearly distinguished.

4. Bias due to deviations from intended interventions

- **Rating: Moderate risk**
- **Rationale**: Four patients dropped out due to treatment-related side effects, which could affect the efficacy assessment. However, this is a relatively small number compared to the overall sample size.

5. Bias due to missing data

- **Rating: Low risk**
- **Rationale**: The study appears to have complete follow-up data for all included patients. The median follow-up duration was 76 months, which is adequate for assessing both short and long-term outcomes.

6. Bias in measurement of outcomes

- **Rating: Low risk**
- **Rationale**: The outcomes (OS, DFS, DMFS) are objective measures not likely subject to detection bias. Follow-up protocols were the same for both groups with standardized imaging at regular intervals.

7. Bias in selection of the reported result

- **Rating: Low risk**
- **Rationale**: The study reported all primary outcomes as stated in the methods section. Both 3-year and 5-year survival rates were presented, and multivariate analyses accounted for potential confounders.

Overall assessment:

Overall risk of bias: Serious risk

The main limitation is the non-randomized design with clear baseline differences between groups, particularly in ECOG performance status, which is a strong prognostic factor. Patients in the UFT group were younger and had better performance status, which likely contributed to their better outcomes independently of the intervention. While multivariate analysis attempted to adjust for confounders, unmeasured factors likely influenced both treatment selection and outcomes. The authors acknowledge that "the strength of evidence and statistical methods in retrospective studies are lower than those in prospectively randomized trials" and suggest that prospective studies are needed to confirm their findings.

ROBINS-I Assessment for Huang et al. (2022)

1. Bias due to confounding

- **Rating: Moderate risk**
- **Rationale**: This was a retrospective comparison between a phase II trial cohort (UFTm) and a non-trial cohort (non-UFTm). While most baseline characteristics were balanced, there were some differences: median number of SCC-involved LNs was higher in non-UFTm (4.0 vs. 2.5, p=0.038) and a trend toward longer radiotherapy duration in non-UFTm (54.6 vs. 50.5 days, p=0.061). Multivariate analysis was conducted to adjust for these potential confounders.

2. Bias in selection of participants

- **Rating: Moderate risk**

- **Rationale**: Only patients who completed CCRT without early relapse (within 2 months) were included, which may have selected for better prognosis patients in both groups. The non-UFTm cohort consisted of patients who met trial eligibility but didn't participate, potentially introducing selection bias. Some selection reasons (patient/physician discretion) were not fully explained.

3. Bias in classification of interventions

- **Rating: Low risk**
- **Rationale**: The intervention (UFTm) was clearly defined as UFT 300-400 mg/day based on BSA for 1 year after CCRT. The treatment protocol and dosing were well-described.

4. Bias due to deviations from intended interventions

- **Rating: Moderate risk**
- **Rationale**: Only 62.5% of patients completed the full UFTm course, with the remaining terminating early due to disease relapse (21.8%), poor compliance (14.1%), or adverse events (1.6%). This substantial non-completion rate may affect the assessment of the true intervention effect.

5. Bias due to missing data

- **Rating: Low risk**
- **Rationale**: The study appears to have complete outcome data for all included patients. The median follow-up of 43 months was adequate for assessing outcomes, and no significant missing data issues were reported.

6. Bias in measurement of outcomes

- **Rating: Low risk**
- **Rationale**: The outcomes (OS, EFS, LRC, DMF) were objective measures with clear definitions. Follow-up protocols were standardized with regular imaging evaluations. There's no indication that outcome assessment was influenced by knowledge of the intervention.

7. Bias in selection of the reported result

- **Rating: Low risk**
- **Rationale**: The study reported all primary outcomes as stated in the methods section. Both univariate and multivariate analyses were presented for all outcomes. There's no evidence of selective reporting based on statistical significance.

Overall assessment:
Overall risk of bias: Moderate risk
The main limitations are the non-randomized design, potential selection bias in the non-UFTm group, and the high rate of incomplete UFTm treatment. However, the study's strengths include balanced baseline characteristics, appropriate multivariate analyses to adjust for potential confounders, standardized outcome assessment, and transparent reporting of results.

ROBINS-I Assessment for Lien et al. (2023)

1. Bias due to confounding

- **Rating: Serious risk**

- **Rationale**: This was a retrospective, non-randomized study where UFT maintenance therapy was decided "at physician's discretion" rather than through randomization. Although baseline characteristics appear balanced between groups, unmeasured confounders likely influenced treatment decisions. Multivariate analysis adjusted for some confounders but cannot account for all potential selection biases in treatment assignment.

2. Bias in selection of participants

- **Rating: Moderate risk**
- **Rationale**: Clear inclusion criteria were established, but the study relied on a retrospective database from a single institution. Patient selection for UFT maintenance was not randomized, and selection for treatment was based on physician preference, which may have resulted in preferential treatment for patients with better prognosis or performance status.

3. Bias in classification of interventions

- **Rating: Low risk**
- **Rationale**: The intervention (UFT maintenance) was clearly defined, as was the biomarker assessment (cortactin expression). The immunohistochemical staining method for cortactin was well described with specific criteria for positivity.

4. Bias due to deviations from intended interventions

- **Rating: Low risk**
- **Rationale**: The study analyzed the effect of UFT maintenance as it was implemented in clinical practice. There is no indication of significant deviation from the intended intervention.

5. Bias due to missing data

- **Rating: Low risk**
- **Rationale**: The study appears to have complete follow-up data with a median follow-up of 65 months. No significant missing data issues were reported.

6. Bias in measurement of outcomes

- **Rating: Low risk**
- **Rationale**: The outcomes (RFS and OS) are objective measures with clear definitions. Follow-up protocols were standardized, and outcome assessment was unlikely to be influenced by knowledge of the intervention.

7. Bias in selection of the reported result

- **Rating: Low risk**
- **Rationale**: The study reported all primary outcomes as specified, with appropriate subgroup analyses by cortactin status. There's no evidence of selective reporting based on statistical significance.

Overall assessment:
Overall risk of bias: Serious risk

The main limitation is the retrospective, non-randomized design with treatment decisions made at physician discretion. While multivariate analysis was conducted to adjust for confounders, unmeasured factors likely influenced both treatment selection and outcomes. The stratification by cortactin expression adds value as a predictive biomarker for UFT benefit, but the findings should be validated in a prospective randomized trial. The authors acknowledge these limitations, noting that "the retrospective and non-randomized study design may be a major bias" and that "further prospective randomized controlled trials with larger cohort are warranted."

ROBINS-I Assessment for Yeh et al. (2021)

1. Bias due to confounding

- **Rating:** Serious risk
- **Rationale:** This retrospective cohort study lacked randomization. Although multivariate analysis adjusted for several clinical and pathological variables (e.g., ENE, LVI, tumor stage), imbalances in baseline characteristics were present—patients receiving UFT had worse prognostic features (more ENE, LVI, and positive margins). The choice to administer UFT was not randomized but likely influenced by perceived risk, leading to potential unmeasured confounding.

2. Bias in selection of participants

- **Rating:** Serious risk
- **Rationale:** Patients were retrospectively selected from a single institution and included based on treatment records and pathology findings. The treatment assignment (UFT vs. control) was influenced by physician judgment rather than predefined eligibility criteria, increasing the likelihood of selection bias. Furthermore, patients with incomplete records or without follow-up were likely excluded, which could further bias the sample.

3. Bias in classification of interventions

- **Rating:** Moderate risk
- **Rationale:** UFT exposure was determined through medical record review. The dose and duration varied (100–400 mg/day for 3–12 months), and while subgroup analyses by duration were performed, the absence of standardized treatment protocol could lead to misclassification or inconsistent treatment documentation.

4. Bias due to deviations from intended interventions

- **Rating:** Moderate risk
- **Rationale:** There was no formal intervention protocol. UFT administration duration varied by patient, and adherence was monitored via outpatient visits, but no strict compliance checks were reported. Patients may have discontinued or altered therapy for reasons not captured systematically.

5. Bias due to missing data

- **Rating:** Low risk
- **Rationale:** The study included all 240 eligible patients, and outcomes were consistently reported. There is no indication of differential follow-up or substantial missing data, and survival analyses were conducted for all included patients.

6. Bias in measurement of outcomes

- **Rating:** Moderate risk
- **Rationale:** While overall survival is an objective measure, disease-free survival and distant metastasis-free survival depend on clinical judgment and imaging interpretation, potentially influenced by knowledge of treatment group. The study was not blinded, which may introduce detection bias.

7. Bias in selection of the reported result

- **Rating:** Low risk
- **Rationale:** All major outcomes (OS, DFS, DMFS, adverse events) were prespecified and reported transparently. Subgroup and duration analyses were also described in detail. There is no indication of selective reporting.

Overall Assessment: Overall risk of bias: Serious risk

The overall evidence is at serious risk of bias, primarily due to the observational design, non-randomized treatment allocation, and potential residual confounding. The stronger prognosis in the UFT group despite worse baseline characteristics supports potential treatment benefit, but causal inference remains limited.

ROBINS-I Assessment for Chen et al. (2019)

1. Bias due to confounding

- **Rating: Serious risk**
- **Rationale**: This retrospective study had physicians prescribe UFT "based on their personal clinical judgment" rather than standardized criteria. While baseline characteristics were balanced, unmeasured confounders likely influenced treatment decisions. The non-randomized design means that while statistical adjustments were made for known variables, systematic differences between groups may persist.

2. Bias in selection of participants

- **Rating: Serious risk**
- **Rationale**: Assignment to intervention vs. control groups was based on physician decisions rather than randomization. Although statistical methods were used to adjust for known variables, the potential for selection bias remains high as participants were selected from a database retrospectively.

3. Bias in classification of interventions

- **Rating: Moderate risk**
- **Rationale**: The intervention was clearly defined (UFT 400mg daily for 1 year), but due to the retrospective design, there may be issues with how interventions were recorded or classified in the medical records.

4. Bias due to deviations from intended interventions

- **Rating: Moderate risk**
- **Rationale**: Five patients dropped out due to adverse effects, which may impact outcomes.

The study does not thoroughly describe methods for monitoring adherence to the intervention protocol.

5. Bias due to missing data

- **Rating: Low risk**
- **Rationale**: The study appears to report data on all included patients with no significant mention of missing data issues.

6. Bias in measurement of outcomes

- **Rating: Moderate risk**
- **Rationale**: While overall survival and disease-free survival are objective endpoints, the assessment of disease recurrence or progression may be influenced by clinicians aware of the patients' treatment status in this non-blinded study.

7. Bias in selection of the reported result

- **Rating: Low risk**
- **Rationale**: The study reported the predetermined primary endpoints (OS, DFS) and safety outcomes.

Overall assessment:
Overall risk of bias: Serious risk
The main limitations are the retrospective design and non-randomized treatment allocation based on physician clinical judgment, which likely introduce confounding and selection bias. While the study attempted to control for known variables through statistical analysis, unmeasured confounding factors cannot be excluded. The authors acknowledge these limitations, noting that "the strength of the evidence and statistical methods in retrospective studies was lower than in prospectively randomized trials" and suggesting that their findings should be confirmed with larger prospective studies.

ROBINS-I Assessment for Liu et al. (2017)
1. Bias due to confounding

- **Rating: Serious risk**
- **Rationale**: The treatment (AdjCT vs. no AdjCT) was not randomized. Although baseline patient characteristics (age, sex, stage, pathology) were statistically similar between groups, substantial confounding exists. Notably, the timing of treatment (pre- vs. post-2004) was strongly associated with both the likelihood of receiving AdjCT and the radiotherapy technique (IMRT vs. 2D/3D RT), as well as exposure to neoadjuvant/concurrent chemotherapy. These confounders were not adequately controlled despite subgroup analyses.

2. Bias in selection of participants

- **Rating: Serious risk**
- **Rationale**: Participants were selected retrospectively from a clinical database spanning 15 years (1994–2009). Many patients who did not receive AdjCT were treated before UFT became reimbursed, introducing time-based selection bias. Additionally, patient inclusion was conditional on completion of curative RT, potentially excluding those with poorer

performance or early relapse.

3. Bias in classification of interventions

- **Rating: Moderate risk**
- **Rationale**: AdjCT was defined as oral UFT for 12 months. Intervention classification was based on medical records, but adherence and exact start date varied, and four patients also received MEP chemotherapy. Variation in treatment exposure reduces clarity and introduces potential misclassification.

4. Bias due to deviations from intended interventions

- **Rating: Moderate risk**
- **Rationale**: There was no standardized protocol for initiating AdjCT (timing and administration were based on clinical judgment and patient condition). While 122 of 154 completed 12 months, others received varied durations or doses. No per-protocol analysis or detailed compliance tracking was reported, and the reasons for dose modifications were not uniformly applied.

5. Bias due to missing data

- **Rating: Low risk**
- **Rationale**: The study reports outcomes for all 403 patients. Follow-up was adequate (median 72 months), and relapse and survival data were complete. No indication of selective loss to follow-up or outcome-dependent missing data.

6. Bias in measurement of outcomes

- **Rating: Moderate risk**
- **Rationale**: Although survival outcomes (OS, PFS, DMFFS) were objectively defined and consistently measured, lack of blinding to treatment group and retrospective ascertainment of relapse could introduce detection bias—especially for outcomes like disease progression or site-specific failure.

7. Bias in selection of the reported result

- **Rating: Low risk**
- **Rationale**: The study appears to report all prespecified outcomes (OS, PFS, DMFFS, NPFFS, NFFS). Subgroup analyses were clearly stated and results were presented transparently, with Kaplan–Meier plots and hazard ratios for all outcomes.

Overall assessment:
Overall risk of bias: Serious risk
Despite a large sample size and long follow-up, the retrospective, non-randomized nature of the study, combined with substantial confounding (especially era-based treatment selection), poses serious limitations to causal inference. Subgroup analyses partially address confounding but do not fully resolve it.

ROBINS-I Assessment for Twu et al. (2014)
1. Bias due to confounding

- **Rating: Serious risk**

- **Rationale**: This was a retrospective, non-randomized study where adjuvant chemotherapy was decided based on "pre-existing chart record" and institutional policy rather than randomization. The treatment allocation was time-dependent (adjuvant group treated after 2005, control group earlier), introducing temporal bias. Despite comparable baseline characteristics, unmeasured confounders likely influenced treatment decisions. Notably, post-RT pEBV DNA levels were significantly higher in the control group (median 174 vs 12 copies/mL, $p<0.0001$), suggesting potential prognostic imbalance at baseline.

2. Bias in selection of participants

- **Rating: Moderate risk**
- **Rationale**: Clear inclusion criteria were established (persistently detectable pEBV DNA after RT), but selection was retrospective from three datasets. The policy for adjuvant treatment changed over time, with metronomic adjuvant chemotherapy being recommended after 2005 due to insurance reimbursement changes. This temporal aspect of selection could bias results, as more recent patients might have received improved overall care.

3. Bias in classification of interventions

- **Rating: Low risk**
- **Rationale**: The intervention (metronomic adjuvant chemotherapy with oral UFT) was clearly defined, as was the biomarker assessment (pEBV DNA). The PCR method for pEBV DNA quantification was consistent across patients.

4. Bias due to deviations from intended interventions

- **Rating: Low risk**
- **Rationale**: The study analyzed the effect of adjuvant chemotherapy as implemented in clinical practice. Treatment adherence was documented with 22 of 33 patients completing the full 12-month oral UFT course. Reasons for non-completion were documented (disease progression in 6, patient refusal in 2).

5. Bias due to missing data

- **Rating: Low risk**
- **Rationale**: The study reports complete follow-up data with a median follow-up of 70 months for surviving patients. No significant missing data issues were reported for the primary outcomes.

6. Bias in measurement of outcomes

- **Rating: Moderate risk**
- **Rationale**: While survival outcomes are objective measures, the restaging workups differed between groups. Only 2 patients in the adjuvant group received FDG-PET scans, which are more sensitive than conventional imaging used for most patients. This differential diagnostic approach could introduce bias in detecting recurrence or metastasis.

7. Bias in selection of the reported result

- **Rating: Low risk**

- **Rationale**: The study reported all primary outcomes as specified (OS, PFS, NPFFS, NFFS, MFS) with appropriate subgroup analyses. The authors conducted additional analyses with matched cohorts to address the imbalance in post-RT pEBV DNA levels, demonstrating transparency in reporting.

Overall assessment: Overall risk of bias: Serious risk
The main limitation is the retrospective, non-randomized design with treatment decisions influenced by temporal policy changes rather than random allocation. The significant imbalance in post-RT pEBV DNA levels between groups is concerning, though partly addressed through matched cohort analysis. The authors acknowledge these limitations, noting that "limitations of the present study include (1) a retrospective design, (2) patients in the AdjCT group being treated after 2005, and (3) most control patients being treated in an earlier period."

Supplement S4: SWiM-Based Critical Appraisal

SWiM-Based Critical Appraisal for Huang et al. (2021)
1. Study Rationale
(1) Clinical Background
Oral cavity squamous cell carcinoma is common and lethal, especially in Taiwan, where many patients present with advanced-stage disease. Standard treatments (surgery + CCRT) are insufficient to prevent high rates of local and distant recurrence. New strategies are needed to improve outcomes.
(2) Problem Statement
Despite definitive surgery and chemoradiation, stage IV oral cancer still has poor disease-free and overall survival due to recurrence and distant metastasis. The clinical benefit of adding metronomic chemotherapy (low-dose, continuous therapy) with UFT has not been well explored in this specific population.
(3) Hypothesis
Adding oral UFT as maintenance metronomic therapy following definitive CCRT can improve disease-free survival, overall survival, and distant metastasis-free survival in non-metastatic stage IV oral cavity cancer patients.
2. What Was Done
(1) Study Design
Retrospective, single-center cohort study conducted between 2008–2014. Ninety-three patients with stage IVa/IVb oral cavity cancer who underwent surgery and adjuvant cisplatin-based CCRT were enrolled.
(2) Treatment Arms

- **UFT group (n = 51):** Post-CCRT metronomic therapy with oral UFT (400 mg/day for 12 months)
- **Non-UFT group (n = 42):** No maintenance therapy post-CCRT

(3) Outcome Measures

- **Primary outcomes:** 3- and 5-year OS and DFS
- **Secondary outcomes:** 3- and 5-year DMFS
- **Toxicity:** Graded adverse events during maintenance UFT treatment

(4) Statistical Analysis

- Survival curves using Kaplan–Meier method
- Log-rank tests for between-group comparisons
- Cox proportional hazards models for univariate and multivariate analysis
- Significance threshold: $p < 0.05$

3. Interpretation
(1) Primary Results

- **3-year OS:** 74.96% (UFT) vs. 48.47% (non-UFT), p = 0.001
- **5-year OS:** 44.64% (UFT) vs. 44.43%, p = 0.016
- **3-year DFS:** 53.05% (UFT) vs. 35.41%, p = 0.011
- **5-year DFS:** 39.57% (UFT) vs. 31.48%, p = 0.018
- **Multivariate analysis:** UFT use independently predicted improved OS (HR 0.37; 95% CI 0.20–0.70; p = 0.003)

(2) Subgroup Analyses

- Significant improvement in DMFS in UFT group
  - 3-year DMFS: 63.73% vs. 42.82%, p = 0.004
  - 5-year DMFS: 42.25% vs. 38.93%, p = 0.02
- N2/N3 nodal status associated with poorer prognosis

(3) Toxicity

- UFT was generally well tolerated

- No grade 4/5 adverse events
- Grade 3 toxicities occurred in <3% of patients
- Anorexia was the only adverse event significantly more frequent in UFT group (28.57% vs. 7.84%, p = 0.012)

4. Meaning

(1) Clinical Implications

Metronomic UFT maintenance therapy may improve long-term outcomes in patients with non-metastatic stage IV oral cavity cancer, potentially delaying recurrence and reducing distant metastases. The treatment is orally administered and relatively well-tolerated.

(2) Relevance to Current Practice

Given the simplicity, affordability, and safety of UFT, this study provides supportive real-world data for its off-label use as maintenance therapy in a high-risk population. However, clinical adoption should be cautious due to the retrospective nature of the evidence.

(3) Limitations

- Retrospective, non-randomized design
- Potential selection bias (younger age and better ECOG status in UFT group)
- Single-center data may limit generalizability
- Small sample size
- Lack of biomarker stratification (e.g., no EBV or genetic profiling)

(4) Future Directions

- Prospective randomized trials are needed to confirm the benefit of metronomic UFT
- Optimal duration and dosing of maintenance therapy should be explored
- Stratification based on molecular or immunologic markers could personalize therapy

SWiM-Based Critical Appraisal for Huang et al. (2022)

1. Study Rationale

(1) Clinical Background

Oral cavity squamous cell carcinoma with pathologic extranodal extension (pENE) is associated with poor prognosis and high risk of distant metastasis despite surgery and adjuvant chemoradiotherapy. Conventional treatment improves local control but not distant failure rates.

(2) Problem Statement

There is a significant unmet need for systemic strategies to reduce distant metastasis in high-risk resected oral cavity squamous cell carcinoma patients with pENE+. Although UFT has demonstrated antitumor and anti-angiogenic properties, its role as maintenance therapy post-CCRT in this population is unclear.

(3) Hypothesis

Maintenance oral UFT after adjuvant CCRT improves overall survival, event-free survival, and reduces distant metastasis in resected pENE+ oral cavity squamous cell carcinoma compared to observation alone.

2. What Was Done

(1) Study Design

Retrospective cohort analysis including patients enrolled in a phase II UFT trial and contemporaneous non-UFT controls who met trial eligibility but did not receive UFT. Inclusion period: March 2015–December 2017.

(2) Treatment Arms

- **UFT group (n=64):** Oral UFT for 1 year post-CCRT
- **Non-UFT group (n=39):** No further therapy post-CCRT
  All patients had resected oral cavity squamous cell carcinoma with pENE and completed CCRT.

(3) Outcomes Measured

- **Primary:** OS, EFS
- **Secondary:** Locoregional control, distant metastasis-free survival
- **Exploratory:** OS in relapsed patients, safety of UFT

(4) Statistical Analyses

- Kaplan–Meier curves + log-rank test
- Cox regression for univariable and multivariable analysis
- Subgroup and sensitivity analyses by lymph node burden and comorbidity index

3. Interpretation

(1) Primary Results

- **2-year distant metastasis rate:** 25.8% (UFT) vs. 44.2% (non-UFT)
- **OS:** HR 0.31 (95% CI 0.17–0.57), $p < 0.001$
- **EFS:** HR 0.45 (95% CI 0.25–0.82), $p = 0.009$
- **DMF:** HR 0.47 (95% CI 0.24–0.95), $p = 0.035$
- **Multivariable analysis:** UFT independently associated with improved OS and EFS

(2) Subgroup Analyses

- Relapse rate: 35.9% (UFT) vs. 56.4% (non-UFT), $p = 0.042$
- Median OS in relapsed patients: 21.0 vs. 11.0 months ($p < 0.001$)
- Oligometastasis surgery was more feasible in UFT (53%) than non-UFT (6%)

(3) Toxicity: Mostly mild; only two grade 3 events (anemia and mucositis)

4. Meaning

(1) Clinical Implications

UFTm appears to improve survival and reduce distant failures in resected pENE+ OSCC following standard adjuvant CCRT. Its favorable toxicity profile and oral route make it an attractive maintenance strategy.

(2) Relevance to Practice

This study supports UFT as a potential maintenance approach for high-risk oral cavity squamous cell carcinoma. However, being retrospective and partially based on a single-arm trial cohort, findings should be validated in randomized trials.

(3) Limitations

- Retrospective design, potential selection bias despite matched eligibility
- Non-randomized comparison between trial participants and non-participants
- Imbalance in some nodal parameters (e.g., SCC-involved nodes)
- Underestimation of early relapse: 17 patients (12.9%) relapsed before UFT initiation

(4) Future Directions

- Prospective randomized trials to confirm efficacy
- Consider perioperative or earlier systemic interventions for ultra-high-risk subgroups
- Exploration of combination strategies with immunotherapy or anti-inflammatory agents

SWiM-Based Critical Appraisal for Lien (2023)

1. Study Rationale

(1) Clinical Background

Hypopharyngeal cancer (HPC) is a rare but aggressive malignancy with a poor prognosis despite standard treatments such as laryngopharyngectomy followed by adjuvant chemoradiotherapy (CRT). Cortactin, a cytoskeletal protein involved in cell motility and invasion, has been identified as a poor prognostic biomarker in several solid tumors but is understudied in HPC. UFT, an oral 5-FU prodrug, is used in some institutions as maintenance therapy post-CRT, although its utility in HPC has not been fully established.

(2) Problem Statement

There is limited evidence regarding the prognostic role of cortactin in HPC, and it is unknown whether cortactin expression can guide the use of UFT maintenance therapy in this population.

(3) Hypothesis

Cortactin overexpression is associated with worse outcomes in HPC, and UFT maintenance therapy after CRT improves survival specifically in patients with cortactin-positive tumors.

2. What Was Done

(1) Study Design

Retrospective, single-center observational study (2007–2015) at E-Da Hospital, Taiwan.

(2) Treatment Arms

- **N = 157** patients with HPC who underwent laryngopharyngectomy followed by CRT for high-risk pathology.
- Cortactin expression assessed by immunohistochemistry.
- Stratified into cortactin (+) vs. (–), and UFT maintenance (+) vs. (–).
- UFT use was based on physician discretion.

(3) Outcomes Measured

- **Primary outcomes:** Recurrence-free survival (RFS) and overall survival

(4) Analysis: Kaplan–Meier survival curves, log-rank tests, and Cox regression multivariate analysis

3. Interpretation

(1) Primary Results

- **Cortactin expression:**
  - Median RFS: 10.2 months (cortactin +) vs. 86.7 months (–), $P < 0.001$
  - Median OS: 16.9 months (cortactin +) vs. 93.4 months (–), $P < 0.001$
- **UFT effect in cortactin (+):**
  - RFS: 13.6 vs. 7.0 months, $P = 0.006$
  - OS: 24.0 vs. 10.0 months, $P < 0.001$
- **UFT effect in cortactin (–):**
  - No statistically significant difference in RFS or OS
- **Multivariate analysis:**
  Cortactin positivity, male gender, betel nut use, and ENE were independent predictors of poor outcome.

(2) Subgroup Analysis

UFT conferred a survival benefit only in cortactin-positive patients. No significant benefit was seen in cortactin-negative patients.

(3) Toxicity

Not specifically reported in this article. The focus was on efficacy and biomarker-stratified outcomes.

4. Meaning

(1) Clinical Implications

Cortactin may be a useful prognostic and predictive biomarker in HPC. UFT maintenance therapy should be considered in cortactin-positive patients after adjuvant CRT. This stratified approach could optimize resource use and therapeutic outcomes in a high-risk population.

(2) Relevance to Practice

Findings suggest a personalized treatment strategy using cortactin expression to guide UFT maintenance. However, practice change should await validation from prospective or randomized trials.

(3) Limitations

- Retrospective, non-randomized design
- UFT allocation by physician choice → risk of selection bias
- Small sample size and single-center data limit generalizability
- Cortactin expression assessed by a single pathologist without standard scoring criteria

(4) Future Directions

- Validation of cortactin as a biomarker in prospective or multicenter studies
- Randomized trials of UFT in cortactin-positive HPC
- Exploration of cortactin-driven biology and interaction with 5-FU-based therapies

SWiM-Based Critical Appraisal for Yeh (2021)

1. Study Rationale

(1) Clinical Background

Head and neck squamous cell carcinoma (HNSCC) frequently presents as locally advanced disease (stage III–IV), carrying high risks of recurrence and distant metastasis. Despite aggressive multimodal treatment, 5-year survival rates remain under 50%.

(2) Problem Statement

Standard treatments fail to control micrometastatic disease. Although metronomic chemotherapy has shown promise in other malignancies, its role as maintenance therapy in locally advanced HNSCC is underexplored.

(3) Hypothesis

Adding oral UFT as metronomic chemotherapy after definitive treatment improves overall survival, disease-free survival, and distant metastasis-free survival in locally advanced HNSCC patients.

2. What Was Done

(1) Study Design

Retrospective observational cohort study (2012–2018) from a single institution in Taiwan.

(2) Treatment Arms

- **UFT group (n = 96):** Patients received oral UFT (100–400 mg/day for 3–12 months) following CRT (± surgery).
- **Control group (n = 144):** Received CRT (± surgery) without UFT.

(3) Outcome Measures

- **Primary outcomes:** OS, DFS, DMFS
- **Secondary analyses:** Treatment duration impact, treatment failure patterns, risk factors for recurrence and survival
- **Safety:** Adverse events by CTCAE v4.0

(4) Statistical Analysis

- Kaplan–Meier survival estimates
- Cox proportional hazards regression (univariate and multivariate)
- Subgroup analysis based on duration of UFT treatment
- Significance threshold: $p < 0.05$

3. Interpretation

(1) Primary Results

- **OS:** Not reached in UFT group vs. 54.1 months in controls ($p = 0.008$)
  - Adjusted HR = 0.57 (95% CI: 0.31–1.05; $p = 0.073$)
- **DFS:** 54.5 vs. 34.4 months; adjusted HR = 0.51 (95% CI: 0.31–0.82; $p = 0.006$)
- **DMFS:** Significant benefit; HR = 0.57 (95% CI: 0.36–0.91; $p = 0.019$)

(2) Subgroup Analyses

- **By duration:** ≥6 months of UFT yielded significantly better OS, DFS, and DMFS compared to shorter durations or control group.
- **Multivariate analysis:** ENE and CRT were independent poor prognostic factors; UFT was a favorable factor for DFS (adjusted HR = 0.51) and showed a strong trend for OS improvement.

(3) Toxicity

- UFT was well tolerated; only grade 1–2 toxicities were reported.
- Most common AEs: nausea (3.8%), vomiting (3.3%), mucositis (2.1%), neutropenia (2.9%).

- No grade ≥3 toxicity observed.

4. Meaning

(1) Clinical Implications

UFT as metronomic maintenance therapy appears beneficial in improving survival and reducing distant metastasis in locally advanced HNSCC. Its safety profile supports integration into clinical practice for high-risk patients post-CRT.

(2) Relevance to Current Practice

This study supports expanding the use of UFT in high-risk, post-treatment HNSCC patients. However, its retrospective design limits definitive conclusions. Prospective randomized trials are needed to standardize indications and duration.

(3) Limitations

- Non-randomized design with potential selection bias
- Baseline imbalance: UFT group had more high-risk features (ENE, LVI, margin positivity)
- Single-center data may limit generalizability
- No quality-of-life assessment

(4) Future Directions

- Prospective, multicenter RCTs comparing UFT vs. placebo or other agents
- Biomarker-guided treatment stratification
- Investigation of combination maintenance regimens (e.g., UFT + immunotherapy)

SWiM-Based Critical Appraisal for Chen (2019)

1. Study Rationale

(1) Clinical Background

Nasopharyngeal carcinoma has a high incidence in Southeast Asia and remains challenging to treat at stage IV without distant metastases. Although concurrent chemoradiotherapy is the standard of care, long-term outcomes are suboptimal due to risk of recurrence and distant metastasis.

(2) Problem Statement

While metronomic chemotherapy has shown benefits in various cancers, there is limited evidence on its efficacy as a maintenance strategy following definitive CCRT in patients with non-distant metastatic TNM stage IV NPC.

(3) Hypothesis

Adding oral UFT as metronomic maintenance therapy following standard CCRT improves overall survival and disease-free survival in patients with stage IV non-distant metastatic NPC.

2. What Was Done

(1) Study Design

Retrospective cohort study from a single tertiary medical center in Taiwan (Tri-Service General Hospital), conducted between 2010 and 2017.

(2) Treatment Arms

- **UFT group (n = 37):** Received oral UFT (400 mg/day) for 12 months after achieving complete remission post-CCRT
- **Non-UFT group (n = 33):** Received no further treatment after CCRT, unless relapse occurred

(3) Outcome Measures

- **Primary outcomes:**
    - 5-year OS and DFS
- **Secondary outcomes:**
    - Univariate and multivariate analysis of prognostic factors
    - Adverse events graded by CTCAE

(4) Statistical Analysis

- Kaplan–Meier method for survival curves

- Log-rank tests for survival comparison
- Cox proportional hazards regression for univariate and multivariate analysis
- Statistical significance set at $p < 0.05$

3. Interpretation

(1) Primary Results

- **5-year OS:** 91.89% (UFT) vs. 57.58% (non-UFT); $p = 0.004$
- **5-year DFS:** 72.97% (UFT) vs. 36.36% (non-UFT); $p = 0.007$
- **Multivariate analysis:** UFT remained an independent predictor of improved OS (HR = 0.215; 95% CI: 0.056–0.831; $p = 0.03$) and DFS (HR = 0.366; 95% CI: 0.161–0.834; $p = 0.02$)

(2) Subgroup Analyses

No formal stratified subgroup analysis beyond multivariate adjustments, but benefits were seen across a generally balanced cohort (no significant difference in age, ECOG status, T/N stage).

(3) Toxicity

- UFT was well tolerated overall
- No grade 4 or 5 toxicities reported
- Grade 3 toxicities were rare (e.g., anorexia, leukopenia, mucositis)
- Five patients discontinued UFT due to side effects

4. Meaning

(1) Clinical Implications

Metronomic UFT added after CCRT significantly improves both OS and DFS in high-risk, non-distant metastatic stage IV NPC patients. The favorable toxicity profile supports its consideration as a maintenance therapy option.

(2) Relevance to Current Practice

The results support integrating biomarker-free, risk-adapted maintenance UFT in clinical practice, especially in settings where plasma EBV DNA monitoring is not feasible. This may offer a pragmatic strategy for resource-limited settings.

(3) Limitations

- Retrospective, single-center design
- Small sample size (n = 70)
- Selection bias possible, as UFT administration was based on physician discretion
- Toxicity capture relied on chart review, which may underestimate frequency

(4) Future Directions

- Prospective randomized trials to confirm efficacy and define optimal duration
- Further exploration of metronomic UFT in biomarker-positive NPC subsets
- Comparative studies of UFT versus other maintenance agents (e.g., capecitabine or immunotherapy)

SWiM-Based Critical Appraisal for Liu (2017)

1. Study Rationale

(1) Clinical Background

Nasopharyngeal carcinoma is a distinct head and neck malignancy with high radiosensitivity and chemosensitivity. Although concurrent chemoradiotherapy improves locoregional control, distant metastasis has emerged as the predominant failure pattern, especially in high-risk patients.

(2) Problem Statement

Prior adjuvant chemotherapy studies showed inconsistent benefits due to toxic regimens, poor compliance, and unselected patient populations. There remains a need to evaluate low-toxicity AdjCT in clinically high-risk NPC patients.

(3) Hypothesis

Oral UFT as a 12-month metronomic AdjCT improves progression-free survival, overall survival, and distant metastasis-free survival in high-risk NPC patients post-curative radiotherapy ± neoadjuvant/concurrent chemotherapy.

2. What Was Done

(1) Study Design

Retrospective cohort study including 403 NPC patients with at least one high-risk feature treated at a single center (1994–2009).

(2) Treatment Arms

- **AdjCT group (n = 154):** Oral UFT (2 caps BID) for 12 months post-RT
- **Control group (n = 249):** No adjuvant treatment post-RT

(3) Outcome Measures

- **Primary:** OS and PFS
- **Secondary:** NPFFS, NFFS, DMFFS, and toxicity of AdjCT

(4) Statistical Analysis

- Kaplan–Meier survival analysis
- Log-rank test for between-group comparison
- Cox proportional hazards models for subgroup and multivariable analysis
- $P < 0.05$ considered significant

3. Interpretation

(1) Primary Results

- **OS:** 5-year OS: 80.5% (AdjCT) vs. 66.7% (control); HR = 1.89, $p = 0.0001$
- **PFS:** 5-year PFS: 70.5% vs. 59.4%; HR = 1.42, $p = 0.0322$
- **DMFFS:** Significant benefit with AdjCT; 82.1% vs. 68.5%; HR = 1.84, $p = 0.0018$
- No significant differences in NPFFS ($p = 0.9611$) or NFFS ($p = 0.7144$)

(2) Subgroup Analyses

- **Era-matched subgroup (post-2005, n = 169):** OS, PFS, and DMFFS still favored AdjCT ($p < 0.05$)
- **Patients with undetectable post-RT plasma EBV DNA (n = 327):** AdjCT improved OS (HR 0.59; $p = 0.0071$) and DMFFS (HR 0.61; $p = 0.0481$)

(3) Toxicity

- Oral UFT well tolerated;
- Grade ≥3 hematologic toxicity was rare (only 1 patient each with grade 3 anemia and leukopenia)
- 122 patients (79%) completed full 12-month regimen

4. Meaning

(1) Clinical Implications

Metronomic oral AdjCT with UFT improves survival and reduces distant metastasis in high-risk NPC patients with minimal toxicity. The findings support its selective use based on clinical risk features.

(2) Relevance to Current Practice

This study demonstrates the feasibility and benefit of oral low-toxicity AdjCT in a well-defined high-risk subgroup, which is especially relevant for centers lacking access to plasma EBV DNA testing or intensive intravenous AdjCT protocols.

(3) Limitations

- Retrospective, non-randomized design
- Confounding due to treatment era (IMRT, CCRT adoption)
- EBV DNA levels available but not incorporated in original treatment assignment
- Potential selection bias due to reimbursement timing (AdjCT reimbursed only after 2004)

(4) Future Directions

- Prospective randomized trials to validate benefit in biomarker-negative and biomarker-positive populations
- Exploration of AdjCT in EBV DNA-guided treatment strategies
- Evaluation of alternative low-toxicity agents or combinations with immunotherapy

SWiM-Based Critical Appraisal for Twu, 2014
1. Study Rationale
(1) Clinical Background
Nasopharyngeal carcinoma (NPC) is highly radiosensitive and chemosensitive. Despite aggressive curative therapy with radiotherapy and chemotherapy, a subset of patients still relapses due to distant metastases. Plasma Epstein-Barr virus (pEBV) DNA is a validated biomarker for prognosis in NPC.
(2) Problem Statement
Although adjuvant chemotherapy (AdjCT) has not demonstrated a clear benefit in unselected NPC patients, it remains unknown whether biomarker-guided AdjCT can improve outcomes in high-risk subgroups, such as patients with persistently detectable pEBV DNA after curative therapy.
(3) Hypothesis
Metronomic oral UFT AdjCT improves survival and reduces distant failure in NPC patients with persistently detectable pEBV DNA after RT ± induction/concurrent chemotherapy.
2. What Was Done
(1) Study Design
Retrospective case-control study using pooled data from three cohorts (n = 625). Eighty-five patients with persistently detectable pEBV DNA one week post-RT were included.
(2) Treatment Arms
- **AdjCT group (n = 33):** Oral UFT (2 caps BID for 12 months), with or without prior MEP IV chemotherapy
- **Control group (n = 52):** No further treatment after RT ± induction/concurrent chemotherapy
- Matching analysis was performed on 33 controls with similar post-RT EBV DNA levels

(3) Outcome Measures
- **Primary:** Overall survival, progression-free survival
- **Secondary:** Distant metastasis-free survival (MFS), nasopharyngeal failure-free survival (NPFFS), neck failure-free survival (NFFS)

(4) Statistical Analysis
- Kaplan–Meier survival estimates
- Log-rank test for group comparisons
- HRs and 95% CIs calculated
- Matching subgroup analysis by post-RT pEBV DNA levels
- Significance: $p < 0.05$

3. Interpretation
(1) Primary Results
- **5-year OS:** 71.6% (AdjCT) vs. 28.7% (control); HR = 0.27; $p < 0.0001$
- **5-year PFS:** 62.9% vs. 28.7%; HR = 0.43; $p = 0.0037$
- **5-year MFS:** 71.9% vs. 34.6%; HR = 0.33; $p = 0.0008$
- Local and regional recurrence rates were similar between groups
- Post-RT pEBV DNA level strongly predicted relapse in controls

(2) Subgroup Analyses
- In matched cohorts (n = 33 per group), AdjCT group still had significantly better OS ($p = 0.0039$) and MFS ($p = 0.0232$)
- Suggests benefits were not solely due to lower residual viral load

(3) Toxicity
- **MEP IV chemotherapy (n = 4):** Notable grade 3–4 myelosuppression

- **Oral UFT (n = 33):** Very mild toxicity overall
    - Only one patient experienced transient grade 3 leukopenia
    - 3 patients required dose reduction
    - 22 completed 12-month therapy; 3 continued beyond 12 months

4. Meaning

(1) Clinical Implications

Biomarker-guided metronomic AdjCT with oral UFT may significantly improve outcomes in high-risk NPC patients with persistent post-RT EBV DNA. This approach personalizes treatment based on real-time disease burden.

(2) Relevance to Current Practice

This study challenges previous conclusions from AdjCT trials by showing that patient selection is critical. It supports a biomarker-based strategy rather than stage-based selection for AdjCT use in NPC.

(3) Limitations

- Retrospective design
- Historical bias: AdjCT patients were treated after 2005; controls mostly before
- Post-RT EBV DNA levels were lower in AdjCT group, though matching analysis was conducted
- Limited PET use for restaging
- Small sample size

(4) Future Directions

- Research needed on the optimal EBV DNA cutoff for AdjCT initiation
- Exploration of alternative regimens or combination strategies for ultra-high-risk patients
